# Sonographic Indicators for Treatment Choice and Follow-Up in Patients with Pleural Effusion

**DOI:** 10.1155/2018/9761583

**Published:** 2018-10-30

**Authors:** Ruza Stevic, Nikola Colic, Slavisa Bascarevic, Marko Kostic, Dejan Moskovljevic, Milan Savic, Maja Ercegovac

**Affiliations:** ^1^Faculty of Medicine, University of Belgrade, Serbia; ^2^Center for Radiology and MRI, Clinical Center of Serbia, Belgrade, Serbia; ^3^Clinic for Thoracic Surgery, Clinical Center of Serbia, Belgrade, Serbia

## Abstract

**Aim:**

The aim of this study was to evaluate the role of thoracic sonography in treatment of pleural effusions and to identify sonographic indicators for surgical intervention.

**Materials and Methods:**

This study included 378 patients with pleural effusions. US characteristics of effusions as the echo structure and pleural thickening were analyzed. Regarding the US finding, the diagnostic or therapeutic procedure was performed.

**Results:**

The study included 267 male and 111 female patients, an average of 56.7 years. Infection was the most frequent cause of effusion. Two hundred sixty-nine patients had loculated and 109 free pleural effusion. Most frequent echo structure of loculated effusion was complex septate, whereas free effusion was mostly anechoic. Successful obtaining of the pleural fluid without real-time guidance was in 88% and under real-time guidance in 99% patients (*p* < 0.012). There was no significant difference in success rate between free and loculated effusion and regarding the echo structure (*p*=0.710 and 0.126, respectively). Complete fluid removal after serial thoracentesis or drainage was achieved in 86% patients. Forty-five patients with significantly thicker pleural peel and impairment of the diaphragmatic function than remaining of the group (*p* < 0.001) underwent surgery. Open thoracotomy and decortication was more frequently performed in patients with completely fixed diaphragm and complex, dominantly septated effusions. There is no significant difference in US parameters comparing to patients underwent VATS, but the number of VATS is too small for valid conclusion.

**Conclusion:**

Thoracic sonography is a very useful tool in the evaluation of clinical course and treatment options in patients with pleural effusions of a different origin.

## 1. Introduction

Chest ultrasonography (US) is a well-established and -validated imaging modality that has been used in diagnosing of pleural effusion, and it also provides highly useful guidance in invasive diagnostic and therapeutic procedures [1–5]. Ultrasound offers a number of advantages over other radiological methods: cost-effective imaging modality without risk of ionizing radiation, the sensitivity for the detection of pleural collection, and providing the examiner with real-time and immediate results. Compared with computed tomography (CT), ultrasound is more sensitive in distinguishing the pleural fluid from pleural thickening and pleural masses and in the detection of thin septations in loculated effusions [2, 3, 6, 7]. Loculated collections pose challenge in the terms of the most efficient treatment selection. Intrapleural septa are of prognostic significance and may predict a more complicated clinical course. The aim of this study is to evaluate the role of thoracic sonography in treatment of pleural effusions, concerning the type and results of different procedures (thoracentesis, chest tube, and surgery), and to identify sonographic indicators for surgical intervention (VATS or open decortication).

## 2. Materials and Methods

### 2.1. Patients

Patients with pleural effusion of a different origin that underwent thoracic ultrasonography in the clinic for thoracic surgery from January 2014 to December 2015 were included in this investigation. Exclusion criteria comprised persons under 18 years, cardiac failure, and postthoracotomy and malignant effusions. Age and gender were recorded as well as the presumed diagnosis at the time of the referral (infectious, malignant, trauma, and others). All patients had chest radiography prior to chest sonography, and the patients with loculated effusion had chest computerized tomography (CT). In all patients who needed invasive procedures, the relevant lab tests were carried out (INR ratio and platelet count). Effusion was classified as left, right, or bilateral.

### 2.2. Sonographic Examination

All US examinations were performed by an experienced radiologist on the ultrasound machine Toshiba Nemio XG using a convex transducer with a frequency of 3.5 MHz. Since loculated fluid collections prevailed in our patients, scanning of the posterior chest wall in the sitting position was performed in most of them. In the bedside patients, the lateral approach was used. At an initial US assessment, the size, echo structure of pleural effusions, and the presence of pleural thickening or nodulations were evaluated. The estimation of the volume of effusion is performed using the qualitative approach: (1) minimal, if an effusion is seen within the costophrenic angle; (2) small, if the effusion is over the costophrenic angle but still within a one-probe range; (3) moderate, if the effusion is greater than a one-probe range but within a two-probe range; and (4) large or massive, if the effusion is bigger than a two-probe range [6]. Effusions were classified as anechoic, complex septate, complex nonseptate, and echogenic [2, 7]. According to the clinical condition, radiological findings, size, and US appearance of fluid collection, the decision about the therapeutic procedure has been made (thoracentesis, chest tube placement, and surgical treatment). The largest and most accessible pocket of fluid for thoracentesis was located, and puncture site, needle direction, and depth of penetration were determined with US. After identification of an appropriate site for thoracentesis, the freehand technique was performed using skin mark for collections where the observation of the needle in real time was not necessary. This nonguided approach was chosen in most patients because it does not require use of a sterile probe cover and an additional assistant. Real-time US-guided thoracentesis was performed in complex loculated and small fluid collections and in the patients in the intensive care unit. Large collections were drained using the standard technique and catheters of 18–20 Fr. The success rate of the invasive procedures was correlated with size, type, and US appearance of collections in all patients. The echo structure of pleural effusion, thickness of pleural peel, and function of the diaphragm was analyzed, and a comparison has been made concerning treatment options.

### 2.3. Statistical Analysis

Statistical analyses were performed using SPSS v.20.0 for Windows software (Chicago IL, USA). Descriptive statistics were used to summarize baseline demographic and clinical characteristics of patients. Results were expressed as mean ± standard deviations for continuous variables and as percentages for categorical variables. Continuous variables were compared by using the T-test. Categorical variables were compared using the chi-square test. Statistical correlation among diaphragm movement and thickness of pleural peel was examined with the ANOVA test. A *p* value <0.05 was considered statistically significant.

## 3. Results

**Figure 1:**
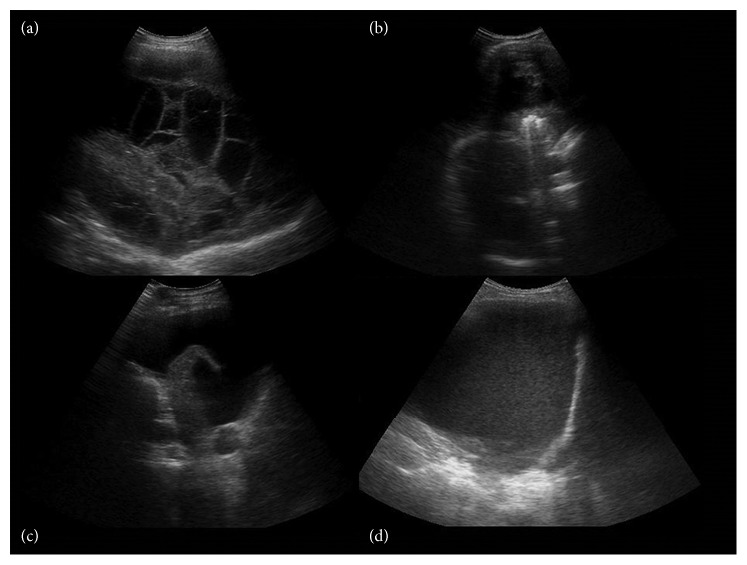
Representative sonographic findings for patients with different pleural effusions. Every chest US demonstrates the inner echo structure from complex septate (a) and nonseptate (b) to anechoic (c) and echogenic (d) pleural effusion.

The study included 267 male and 111 female patients, an average of 56.7 ± 16.7 years (18–87). Infection was the most common cause of effusion followed by other causes. Two hundred sixty-nine patients had loculated and 109 free-flowing effusions (Table 1). Most frequent echo structure of loculated effusion was complex septate (Figure 1), whereas free effusion were mostly anechoic (Figure 1). After the initial US examination, 125 cases were estimated as unsuitable for thoracentesis because of either small amount of pleural fluid or complex effusion with pleural peel. Thoracentesis was indicated in 253 cases. The “free-hand” technique after the skin mark was performed in 173 patients, and thoracentesis under real-time US guidance was done in 80 patients. Successful obtaining of the pleural fluid was 92% (88% for thoracentesis or drainage without real-time guidance and 99% under real-time US guidance) (*p* < 0.012). Twenty of 21 unsuccessful thoracentesis was in small collection. There was no significant difference in success rate between different echo structures although the highest success rate was achieved in anechoic collections (*p*=0.126) (Table 2). In 232 patients, repeated thoracentesis or drainage was performed as a therapeutic choice. Complete fluid removal after serial thoracentesis or drainage was achieved in 200 of 232 (86%) patients. More than half of successfully drained collections had the complex echo structure (Table 3). In twenty-seven out of 32 patients with incomplete fluid evacuation, effusions were either complex nonseptate with thick fluid or with more thickened septations.

**Figure 2:**
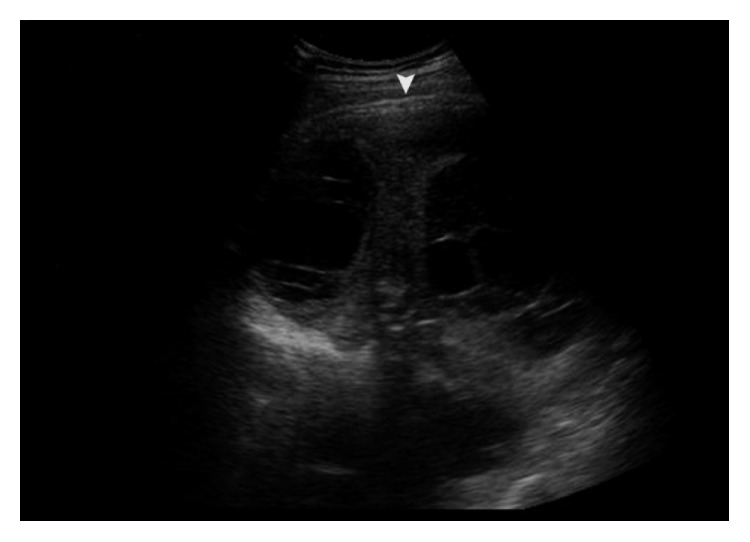
Chest sonography shows smooth pleural thickening (the arrow head) in complex septate effusion.

Pleural thickness in all groups was 5.6 ± 1.5 mm with a significant difference between surgical treated and untreated group. The fixed diaphragm was dominantly found in loculated, complex septate collections and pleural peel thickness more than 6 mm (*p* < 0.001 both) (Figure 2).

Surgery was performed in 45 patients with a statistically significant difference in thickness of pleural peel and diaphragmatic dysfunction compared to the remaining of the group (Table 3). Open thoracotomy and decortication was more frequently performed in patients with completely fixed diaphragm and complex, dominantly septated effusions. VATS has been suitable in patients with free effusion, impaired diaphragm movement, and pleural peel thickness less than 6 mm. There is no significant difference in US parameters between these two groups, but the number of VATS is too small for valid conclusion (Table 4). Surgery was not offered to patients with significant comorbidities, pulmonary embolism, and systemic diseases, regardless of characteristics of effusion, peel, and diaphragm. A number of patients did not give consent for surgical treatment.

## 4. Discussion

Chest sonography is a very convenient tool for evaluation of patients with pleural effusion in assisting thoracentesis and follow-up evaluation and in assessing treatment efficacy. It is well known that US is superior to radiography not only for assessing pleural effusion but also for diaphragm function. This study describes the role of sonography in the evaluation of pleural effusions regarding possible treatment options. Thoracentesis is usually the first diagnostic/therapeutic procedure in patients with established pleural effusion. Overall success rate of thoracentesis was high, and real-time US-guided thoracentesis was superior to the nonguided, skin mark technique. The main causes for thoracentesis failure in 8% patients were small collections with a complex echo structure and procedure without real-time monitoring. Lower success rate in thoracentesis or drainage without real-time guidance can be simply explained by shifting of both fluid collection and skin mark with a change in the patient's position. During the real-time US-guided procedure, constant monitoring of the aspiration site helps avoiding thick septa or efficient disruption of thinner septa which enables successful fluid aspiration [8–14]. More than two-thirds of all patients had loculated effusion with the complex septate echo structure in half of them. This study and literature data confirmed that existence of intrapleural septa may predict a more complicated clinical course [6, 8–10, 15–20]. Our results and reported series show that the smaller number of complications and high success rate of thoracentesis even in complex loculated collections can be explained by the particular choice of the point of puncture in the skin mark-based method and the constant view and following of the needle during the real-time US-guided intervention [13, 14, 16, 21, 22, 23, 24, 25]. Cases with small or thick fluid collection (solid-like appearance) with the complex echo structure and thick septations were evaluated as nondrainable, so inefficient chest tube placement with possible complications has been avoided [10, 18, 26]. Failure of thoracentesis and/or drainage requires surgical treatment in selected patients. Results of this study concerning success rate of thoracentesis and drainage are in accordance with analyzed data that reported success rate from 62% to 97% depending on the echo structure of collection [11, 16, 23]. Preprocedural US can predict the likelihood of success of thoracentesis or drainage but not the patient outcome.

In the analyzed group of surgically treated patients, indication for surgery was based on diaphragmatic function, thickness of pleural peel, and characteristics of pleural fluid, in that order. Accurate identification of US predictors for open thoracotomy vs VATS in this study is limited by small number of patients treated with VATS procedures and requires further investigation on a larger sample size. However, preoperative US is a very useful tool in estimation of pleural peel thickness and diaphragmatic function and may help in planning the type of the procedure and predicting the length of surgery. US evaluation can be used repeatedly during patient treatment without possible risk of ionization associated with CT scan.

## 5. Conclusion

Thoracic sonography is a very useful tool in the evaluation of clinical course and treatment results in patients with pleural effusions of a different origin. Sonographic evidence of septa, pleural peel, and impair of diaphragm movement may indicate the need for surgical intervention. Indication for surgery and estimation of type of surgery (open vs VATS) may be based on serial US examinations with the high accuracy.

## Figures and Tables

**Table 1:** Characteristics of patients.

*Sex*	Male (267)	Female (111)	
*Age*	57 ± 15.6	56.5 ± 18.3	
*Diagnosis*			Total
Empyema	116	39	155
Parapneumonic effusion	61	30	91
Trauma	40	11	51
Others^*∗*^	50	31	81
*Type of effusion*			Total
Loculated	185	84	269
Free	82	27	109
*Echo structure*			Total
Complex septate	104	39	143
Complex nonseptate	48	26	74
Echogenic	35	19	54
Anechoic	80	27	107
*Treatment*			Total
Thoracentesis and chest tube	177	76	253
Surgery	37	8	45

^*∗*^Systemic autoimmune diseases, after cardiosurgery, pulmonary embolism, and abdominal pathology.

**Table 2:** Success rate of thoracentesis regarding the type, echo structure, and size of effusions.

		Unsuccessful		Successful		Total	Sign
		Number	%	Number	%		
Type of effusion	Loculated effusion	18	9	185	91	203	0.710
	Free-flowing effusion	3	6	47	94	50	
Total		21	8	232	92	253		
		Number	%	Number	%	Number	
Thoracentesis	Real-time US-guided thoracentesis	1	1	79	99	80	*p* < 0.012
	Without real-time guidance	20	12	153	88	173	
Total		21	8	232	92	253	
		Number	%	Number	%	Number	
Echo structure	Complex septated	11	10	104	90	115	0.126
	Complex nonseptated	4	7.5	49	92.5	53	
	Echogenic	5	16	26	84	31	
	Anechoic	1	2	53	98	54	
		Number	%	Number	%	Number	
Size of effusion	Small	20	24	65	76	85	*p* < 0.001
	Medium	1	2	111	98	112	
	Large effusion	0	0	56	100	56	

**Table 3:** US characteristics of surgically and nonsurgically treated patients.

Treatment option		Diaphragm function			
		Fixed	Impaired movement	Normal movement	Total
Nonsurgically		49 (15 %)	188 (57 %)	93	330 (100%)
Surgically		15 (33 %)	28 (62%)	2	45 (100%)
Total		64	216	95	375
		*p* < 0.001			

	Echo structure				
Treatment option	Complex septate	Complex nonseptate	Echogenic	Anechoic	Total

Medicamentous	34	22	25	52	133
Drainage/thoracentesis	91	38	23	48	200
Surgically	18	14	6	7	45
	143	74	54	107	378
	*p*=0.090				

	Wall thickness				
Treatment option	*N*		Mean		Standard error mean

Nonsurgically	333		5.39 ± 1.45		0.080
Surgically	45		7.04 ± 1.44		0.215
*p* < 0.001					

**Table 4:** US characteristics of surgically treated patients (open thoracotomy vs VATS).

Treatment option		Diaphragm function			
		Fixed	Impaired movement	Normal movement	Total
Open thoracotomy		15 (37.5%)	24 (60%)	1 (2.5%)	40 (100%)
VATS		0 (0.0%)	4 (80%)	1 (20%)	5 (100%)
Total		15	28	2	45
		*p*=0.059			

	Echo structure				
Treatment option	Complex septate	Complex nonseptate	Echogenic	Anechoic	Total

Open thoracotomy	17 (42.5%)	13 (32.5)	6 (15%)	4 (10%)	40 (100%)
VATS	1 (20%)	1 (20%)	0 (0%)	3 (60%)	5 (100%)
	18	14	6	7	45
	*p*=0.075				

	Wall thickness				
Treatment option	*N*		Mean		Standard error mean

Open thoracotomy	40		7.25 ± 1.4		0.220
VATS	5		5.4 ± 0.55		0.245
*p*=0.006					
